# Unrecognised Second-Trimester Pregnancy With Fetal Demise Presenting as Acute Abdomen and a Negative Pregnancy Test

**DOI:** 10.7759/cureus.98517

**Published:** 2025-12-05

**Authors:** Marwa E Desouky

**Affiliations:** 1 Obstetrics and Gynaecology, Northampton General Hospital, Northampton, GBR

**Keywords:** abdominal pain, fetal demise, intrauterine fetal demise, negative pregnancy test, unrecognised pregnancy, urine β-hcg

## Abstract

A woman in her early 20s presented with abdominal pain and a heavy period, unaware that she was 18 weeks pregnant. Despite a negative urine pregnancy test, imaging revealed intrauterine fetal demise. This rare case highlights the importance of maintaining clinical suspicion and performing early imaging, as pregnancy tests may yield false-negative results in cases of miscarriage and fetal demise. Human chorionic gonadotropin (hCG) is produced by the placenta, but when a pregnancy fails to develop normally, such as in miscarriage, hCG levels may be lower than expected or decline, leading to negative test results despite advanced gestation.

## Introduction

Second-trimester fetal demise is typically associated with well-recognised clinical features such as cessation of fetal movements, abdominal distension, amenorrhoea, and persistently positive pregnancy tests. Pregnancy detection usually relies on the presence of β-human chorionic gonadotropin (β-hCG), which remains elevated throughout gestation. However, unrecognised or “cryptic” pregnancies pose a diagnostic challenge, particularly when patients continue to experience bleeding resembling menstruation or present with nonspecific symptoms, such as abdominal pain, leading clinicians to initially consider more common gynaecological conditions [1].

Although uncommon, false-negative urine or serum β-hCG tests have been reported in the literature, even in cases of advanced pregnancy or trophoblastic disease. One well-documented cause is the “hook effect,” in which extremely high β-hCG concentrations saturate the antibodies in the immunoassay, preventing proper complex formation and resulting in a falsely negative result [2,3]. Case reports describe patients with complete molar pregnancies who had repeatedly negative urine pregnancy tests, with diagnosis only confirmed after quantitative serum β-hCG testing with dilution [3]. Another case involved an adolescent presenting with abdominal pain and bleeding with a negative urine test; subsequent evaluation revealed a molar pregnancy following serum β-hCG measurement and imaging [3].

In this report, we describe a 24-year-old woman who presented with abdominal pain and what she believed to be a heavy menstrual period, later found to have a second-trimester fetal demise despite repeatedly negative urine β-hCG testing. This case emphasises the need to maintain a broad differential diagnosis in reproductive-aged women presenting with abdominal pain or abnormal bleeding, even when qualitative pregnancy tests are negative, and highlights the limitations of urine β-hCG assays in atypical presentations [1,2].

## Case presentation

A woman in her early 20s, P2, presented to the emergency department with heavy per vaginal bleeding and lower abdominal pain. Her obstetric history included two previous lower-segment caesarean sections, with no other significant medical or surgical history. She reported regular menstrual cycles and stated that she was on day 6 of what she believed to be her current menstrual period.

**Figure 1:**
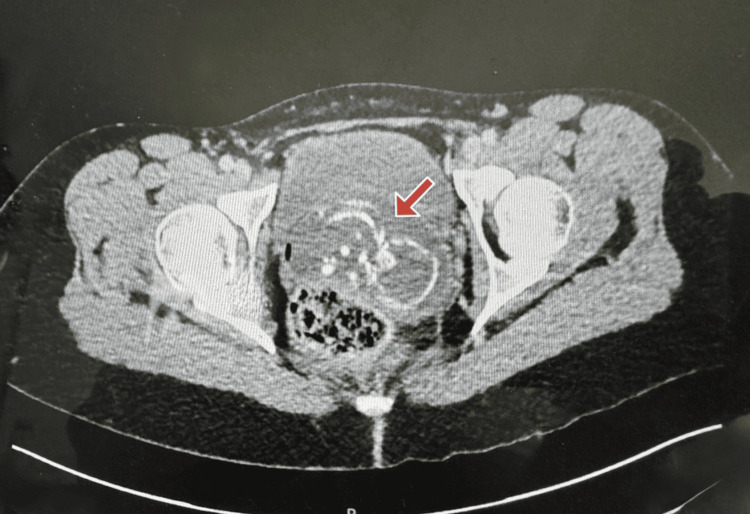
CT scan of the pelvis showing fetal bones within the uterine cavity

Initial investigations in the emergency department demonstrated a negative urine pregnancy test. Due to the severity of her abdominal pain and concern for possible intra-abdominal pathology, a CT scan of the abdomen and pelvis was performed. The scan unexpectedly revealed the presence of fetal bones within the uterus, raising suspicion for an unrecognised pregnancy or intrauterine fetal demise (Figure 1).

**Figure 2:**
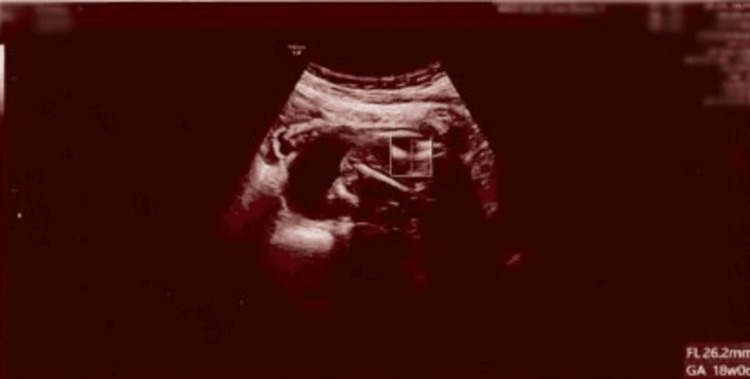
Transabdominal ultrasound demonstrating intrauterine fetal demise, with femur length of approximately 18 weeks

The patient was referred promptly to the gynaecology team for further assessment. On examination, she was haemodynamically stable but continued to report worsening abdominal pain. A repeat urine pregnancy test remained negative, while serum β-hCG measured 80 IU/L [1,2]. A bedside transabdominal ultrasound confirmed the presence of an intrauterine fetus with no detectable cardiac activity. The femur length corresponded to approximately 18 weeks’ gestation, and the fetal anatomy appeared collapsed, consistent with intrauterine fetal demise (Figure 2).

The patient was admitted for further management. The following morning, she experienced a spontaneous complete miscarriage while on the ward. She remained haemodynamically stable throughout and did not require surgical intervention.

## Discussion

This case highlights several diagnostic and clinical challenges associated with unrecognised pregnancy and second-trimester fetal demise. The patient, unaware of her pregnancy at 18 weeks’ gestation, presented with lower abdominal pain and what she believed to be a heavy menstrual period. Remarkably, her urine pregnancy tests were repeatedly negative, and serum β-hCG was low (80 IU/L), despite the advanced gestation [1,2]. Such discrepancies are rare but have been described in the literature, often due to the natural decline of β-hCG following placental demise or assay limitations, including the hook effect, which may produce false-negative results in standard immunoassays [2,3].

Cryptic pregnancies, where a patient remains unaware of gestation, can persist into the second trimester, though this is uncommon. Previous case reports describe patients with advanced gestations who presented with abdominal pain, bleeding, or atypical menstrual patterns and had negative or unexpectedly low pregnancy tests [1-3]. Our case adds to this body of evidence, highlighting that clinicians should consider pregnancy in reproductive-aged women presenting with abdominal pain or abnormal bleeding, even when initial tests are negative.

Imaging played a critical role in the diagnosis. While ultrasound is the standard first-line investigation in suspected pregnancy complications, CT imaging is rarely employed due to radiation concerns. In this case, a CT scan performed for the evaluation of abdominal pain incidentally revealed fetal bones, prompting urgent gynaecological referral [1-3].

Management of intrauterine fetal demise in such cases depends on the patient’s stability, gestational age, and clinical context. Our patient experienced a spontaneous complete miscarriage without the need for surgical intervention. This aligns with previously reported cases, where conservative management was feasible in haemodynamically stable patients [1-3]. Nonetheless, timely recognition of fetal demise is essential to counsel patients appropriately and prevent potential complications such as infection or haemorrhage.

## Conclusions

This case illustrates a rare but important diagnostic pitfall: advanced unrecognised pregnancy with fetal demise and negative pregnancy tests. It underscores the value of clinical suspicion, thorough history-taking, and prompt use of ultrasound in women of reproductive age presenting with abdominal pain and bleeding. Maintaining a broad differential diagnosis can prevent missed or delayed identification of significant obstetric conditions.

## References

[1] Londero AP, Orsaria M, Grassi T (2013). Placental hCG immunohistochemistry and serum free-beta-hCG at 11-13 weeks' gestation in intrauterine fetal demise. Histochem Cell Biol.

[2] Herskovits AZ, Chen Y, Latifi N, Ta RM, Kriegel G (2020). False-negative urine human chorionic gonadotropin testing in the clinical laboratory. Lab Med.

[3] Nigam A, Kumari A, Gupta N (2014). Negative urine pregnancy test in a molar pregnancy: is it possible?. BMJ Case Rep.

